# Retraction note: Protective and antidiabetic effects of extract from *Nigella sativa* on blood glucose concentrations against streptozotocin (STZ)-induced diabetic in rats: an experimental study with histopathological evaluation

**DOI:** 10.1186/s13000-016-0571-6

**Published:** 2016-11-02

**Authors:** Samad Alimohammadi, Rahim Hobbenaghi, Javad Javanbakht, Danial Kheradmand, Reza Mortezaee, Maryam Tavakoli, Farshid Khadivar, Hamid Akbari

**Affiliations:** 1 Department of Physiology, Faculty of Veterinary Medicine, Tehran University, Tehran, Iran; 2 Department of Pathology, Faculty of Veterinary Medicine, Urmia University, Urmia, Iran; 3 Department of Pathology, Faculty of Veterinary Medicine, Tehran University, Tehran, Iran; 4 Faculty of Medicine MD, Graduate Student of Islamic Azad University of Mashhad, Mashhad, Iran; 5 Young Researchers Club and Elites, Mashhad Branch, Islamic Azad University, Mashahd, Iran; 6 Graduate Faculty of Veterinary Medicine, Urmia University, Urmia, Iran; 7 Faculty of Veterinary Medicine, Tehran University, Tehran, Iran; 8 Department of Clinical Science, Faculty of Veterinary Medicine, Urmia University, Urmia, Iran

## Retraction

The Editor-in-Chief and Publisher have retracted this article [1] because the scientific integrity of the content cannot be guaranteed. An investigation by the Publisher found it to be one of a group of articles we have identified as showing evidence suggestive of attempts to subvert the peer review and publication system to inappropriately obtain or allocate authorship. This article showed evidence of plagiarism (most notably from the articles cited [2–5]) and peer review and authorship manipulation.
